# Combined cryotherapy and topical imiquimod, 5-fluorouracil, and tazarotene effectively treats locally advanced basal cell carcinoma and is associated with nearby nodular basal carcinoma regression

**DOI:** 10.1016/j.jdcr.2025.01.015

**Published:** 2025-02-05

**Authors:** William J. Nahm, Olivia L. Shen, Robert S. Kirsner, Christopher A. Mathe, Odysseas V. Nikas, Flor D. Valadez, Evangelos V. Badiavas

**Affiliations:** a New York University Grossman School of Medicine, New York, New York; b University of California, Davis, Davis, California; c Dr. Phillip Frost Department of Dermatology & Cutaneous Surgery, University of Miami Miller School of Medicine, Miami, Florida; d Sylvester Comprehensive Cancer Center, Miami, Florida; e California University of Science and Medicine, Colton, California; f Northeastern University, Boston, Massachusetts; g Shen Dermatology, Temecula, California

**Keywords:** 5-fluorouracil, abscopal effect, bystander effect, cryotherapy, imiquimod, keratinocyte carcinoma, locally advanced basal cell carcinoma, tazarotene, topical therapy

## Introduction

Locally advanced basal cell carcinomas (laBCCs) are characterized by their considerable size, aggressive growth patterns, or recurrent nature. These neoplasms invade adjacent structures, including osseous tissue, cartilage, neural elements, and muscle. A more objective definition for laBCC is the fulfillment of at least one of the following criteria: T2 classification or higher (tumor size ≥2 cm or exhibiting deep invasion) or node-positive status as determined by clinical examination or radiologic assessment.^1^ Treatment approaches for laBCCs include surgical removal with Mohs surgery, radiation therapy, hedgehog pathway inhibitors, and immune checkpoint inhibitors.^2^ The combination of imiquimod, 5-fluorouracil, and retinoids with cryotherapy has proven to be an effective treatment for keratinocyte carcinomas (KCs).^3^ Herein, we present a case of a laBCC on the superior nasal dorsum treated with a direct course of cryotherapy with imiquimod, 5-fluorouracil, and tazarotene application. Also, this combined course promoted a concurrent resolution of a nearby untreated BCC on the left nasal tip.

## Case report

**Fig 1:**
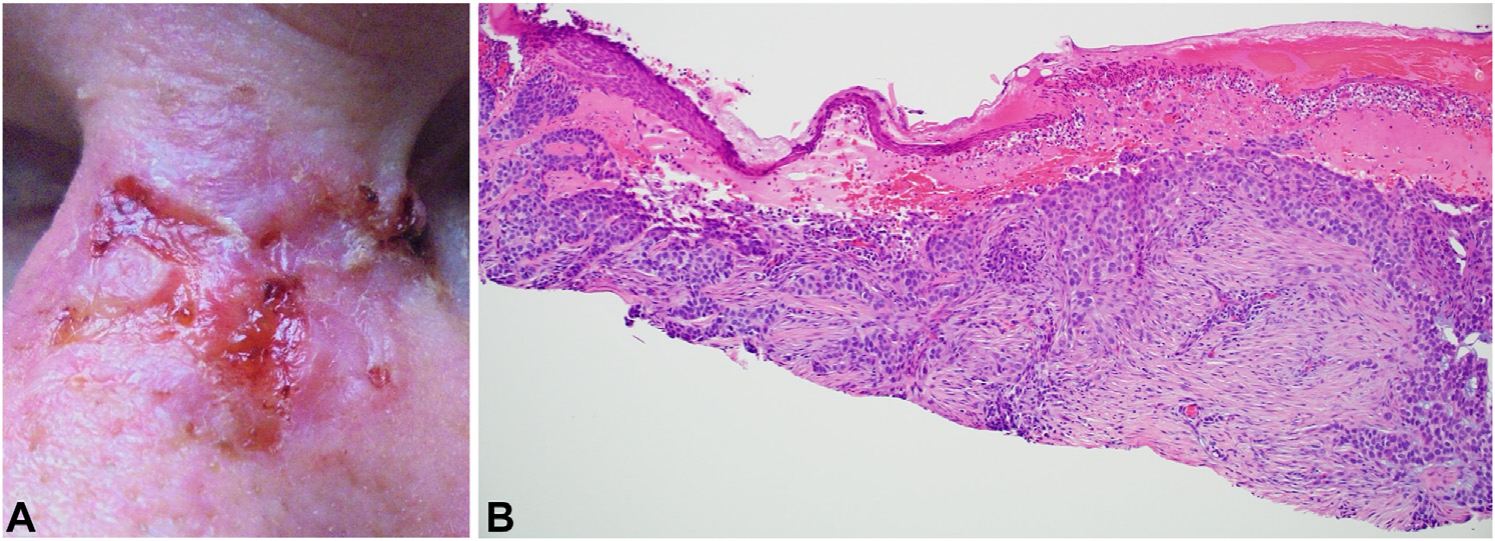
Pretreatment clinical and pathologic images of a morpheaform locally advanced basal cell carcinoma (laBCC) on the nasal dorsum. **A,** Baseline image of a morpheaform laBCC on the nasal dorsum. **B,** Pathology of the nasal dorsum revealed a sclerotic dermis with narrow nests and strands and an eroded epidermis with acutely inflamed crust demonstrating a morpheaform BCC. (Hematoxylin-eosin stain; original magnification: ×200)

**Fig 2:**
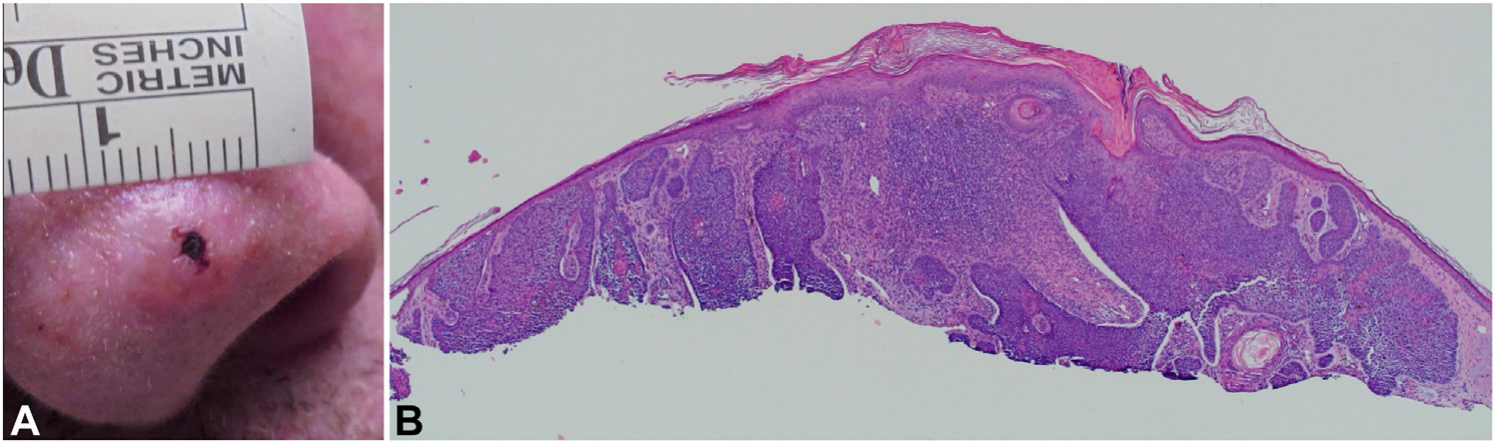
Pretreatment clinical and pathologic images of a nodular basal cell carcinoma (BCC) on the left side of the nasal tip. **A,** Baseline image of nodular BCC on the left side of the nasal tip. **B,** Pathology of the left side of the nasal tip revealed nests and strands of atypical basaloid cells from the undersurface of the epidermis, demonstrating a nodular BCC. (Hematoxylin-eosin stain; original magnification, ×40)

A 60-year-old male presented with a 3.4 cm × 2.6 cm ulcerated plaque on the superior nasal dorsum that had been enlarging over 18 m (Fig 1, *A*). A biopsy revealed a sclerotic dermis with narrow nests and strands and an eroded epidermis with an acutely inflamed crust, delineating a morpheaform BCC (mBCC) (Fig 1, *B*). The tip of the nose presented with a 1.0 cm × 0.9 cm nodular BCC (nBCC) (Fig 2, *A*), which was also confirmed with a biopsy (Fig 2, *B*). There was a 3.2 cm distance from the lower margin of the laBCC to the superior margin of the nBCC. The patient lacked insurance and, after exploring out-of-pocket cost options, declined Mohs surgery and hedgehog pathway inhibitors.

**Fig 3:**
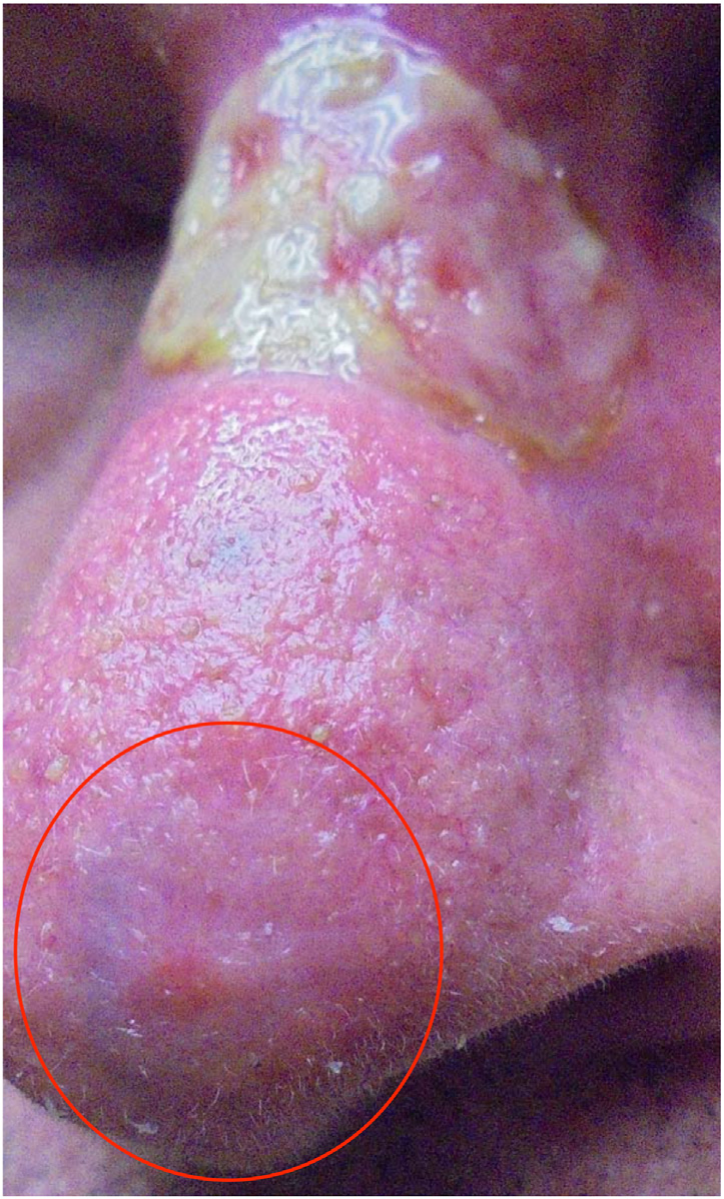
At the end of the 6-week treatment course with cryotherapy and imiquimod/5-fluorouracil/tazarotene on the nasal dorsum, necrotic ulceration with inflammation is seen on the nasal dorsum. In addition, mild inflammation is seen on the untreated nasal tip (red circle).

The patient was then presented with a course of imiquimod, 5-fluorouracil, and tazarotene with biweekly treatments of cryotherapy. The patient was instructed to apply the medications to the laBCC 5 days a week for 6 weeks. Specified amounts of the medications included 1/2 a packet of imiquimod 5% cream, one drop of 5-fluorouracil 2% solution, and 1/2 a pea-sized quantity of tazarotene 0.1% cream. The medications were combined on a bandage and placed on the mBCC overnight. Adjunctive cryotherapy was administered at baseline and biweekly intervals throughout the treatment course, totaling four sessions. Each cryotherapy application lasted 2 seconds and encompassed the lesion with an additional 1-2 mm circumferential margin. The patient developed erythema, swelling, ulceration, and necrotic tissue on the treated lesion site with pain and discomfort, which lasted several weeks during the treatment (Fig 3). Coincidentally, the nBCC on the nasal tip, which was untreated, also experienced some inflammation but no ulceration (Fig 3). This nBCC was not treated because the patient did not feel he could adequately tolerate the side effects and minimize the adverse appearance if two sites were treated simultaneously.

**Fig 4:**
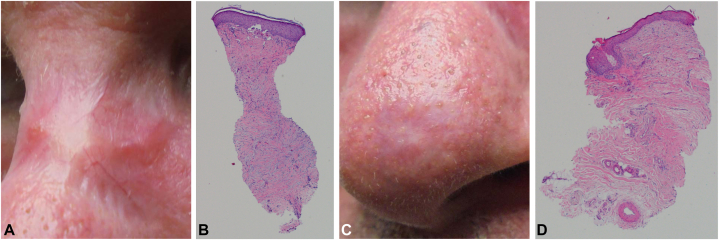
Clinical and histopathological images 6 months post-treatment. **A,** Nasal dorsum with sclerotic plaque. **B,** Histopathology of one of six punch biopsies in this area. All punch biopsies revealed only dermal cicatrix and no evidence of morpheaform basal cell carcinoma (BCC). **C,** Nasal tip with an erythematous shiny patch. **D,** Histopathology of one of two punch biopsies on the nasal tip. Both punch biopsies demonstrated clearance of the nodular BCC. (**B** and **D**, Hematoxylin-eosin stain; original magnification: **B**, ×40 **D**, ×40)

Six months after treatments, six punch biopsies were performed on the superior nasal dorsum where the treatments were administered; all six pathology specimens revealed only dermal cicatrix (Fig 4, *A* and *B*). Two punch biopsies were also performed on the area where the untreated nBCC was present on the tip of the nose, revealing no evidence of cancer (Fig 4, *C* and *D*). Eighteen months postdiagnosis, both sites on the nose were clinically clear of any neoplasm.

## Discussion

Although there are effectual treatment options for laBCC, the courses of cryotherapy and applications of topical combinations of imiquimod, 5-fluorouracil, and tazarotene represent a novel yet cost-effective treatment option.^3^ Previously, topical therapies have been shown to be effective in treating superficial, non-high-risk KCs. With low-risk superficial and nBCCs, topical imiquimod and 5-fluorouracil are recognized for their successful treatment, while topical diclofenac and ingenol mebutate have demonstrated limited efficacy.^4^, ^5^, ^6^ A cryoimmunotherapy treatment protocol (two 5-s cycles of cryotherapy, followed by a 6-week course of imiquimod) was used to treat superficial BCCs.^7^ There is a case report that describes the successful treatment of extensive, radiation-induced Bowen's disease on the hands with nonsequential and combined treatments of imiquimod, tazarotene, and 5-fluorouracil creams.^8^ Moreover, previous studies on combining imiquimod, 5-fluorouracil, and tretinoin showed efficacy against BCCs and SCCs.^3^ While topical combinations of imiquimod, 5-fluorouracil, and tretinoin have shown effectiveness in treating mBCC, multivariate analysis revealed that the high-risk morpheaform subtype demonstrated lower clearance rates compared to other BCC variants.^3^

In this protocol, tazarotene 0.1% cream was substituted for tretinoin 0.1% cream due to the high-risk nature of the mBCC. Although unpublished, in the authors' experience, using tazarotene 0.1% cream instead of tretinoin 0.1% cream in combination had greater efficacy against KCs that were aggressive or larger than 1.0 cm.

The clearance of the untreated nBCC suggests a potential abscopal effect (AbE) or bystander effect (ByE). The AbE was initially described in advanced and metastatic cancers with radiation therapy. The AbE denotes a systemic response to localized treatment, causing regression of distant, untreated tumor lesions. In contrast, the ByE involves local reduction of untreated cancer cells near treated ones, mediated by secreted factors, gap junctions, or cytokine alterations.^9^ Both phenomena represent nontargeted effects of immunogenic cell death, differing in spatial domains.^9^ Evidence of this type of immunogenic-mediated process was also seen when field chemoprevention with a combination of imiquimod, 5-fluorouracil, and tretinoin on the face was seen with lower odds of having KCs in nontreated areas 1 year after field treatment.^10^ Despite applying the combined topicals under a bandage only on the nasal dorsum, we cannot exclude the possibility that the medications could have migrated, been spread inadvertently, or distributed to a distant site through transdermal absorption. As there was a distance of 3.2 cm between the two lesions, these possibilities could have contributed to the regression of the nasal tip BCC.

This case demonstrates the potential efficacy of combining cryotherapy with topical imiquimod, 5-fluorouracil, and tazarotene for treating a laBCC, warranting an investigation into this treatment approach for patients with limited access to conventional therapies or as an alternative to more invasive procedures. The observed clearance of an untreated nBCC suggests a possible antitumor immune response or AbE/ByE.

## Conflicts of interest

None declared.
